# Pulpitis Transiently Affect Hepatic Bone Morphogenetic Protein 9 Expression by Lipopolysaccharide

**DOI:** 10.1016/j.identj.2026.109435

**Published:** 2026-02-19

**Authors:** Tianzhu Song, Dongzhe Song, Yanglin Zeng, Jingli Zhu, Xiangfen Li, Dingming Huang

**Affiliations:** a State Key Laboratory of Oral Diseases & National Center for Stomatology & National Clinical Research Center for Oral Diseases, West China Hospital of Stomatology, Sichuan University, Chengdu, China; b Department of Conservative Dentistry and Endodontics, West China Hospital of Stomatology, Sichuan University, Chengdu, China; c Department of Pediatric Dentistry, West China Hospital of Stomatology, Sichuan University, Chengdu, China; d Key Laboratory of Oral Diseases of Gansu Province Key & Laboratory of Stomatology of State Ethnic Affairs Commission, Northwest Minzu University, Lanzhou, Gansu, China; e Department of Periodontics, Hospital of Stomatology, Lanzhou University, Lanzhou, Gansu, China

**Keywords:** Pulpitis, BMP9, Lipopolysaccharide, hepatic stellate cells, H3K9 dimethylation

## Abstract

**Introduction and aims:**

The connection between oral and systemic diseases is receiving increasing attention. Pulpitis is an infectious disease, and its potential impact on the systemic system deserves further investigation.

**Methods:**

A rat/mouse pulpitis model was utilized to explore the variations in bone morphogenetic protein 9 (BMP9) and lipopolysaccharide (LPS) concentrations within liver tissue and blood. Hepatic stellate cells were cultivated and subsequently stimulated with LPS. Histone H3K9 methylation and associated methyltransferase levels were quantified. G9a function was investigated by the G9a methyltransferase inhibitor UNC0642.

**Results:**

In rats and mice with pulpitis, BMP9 expression initially declined in both the liver and blood, followed by a subsequent increase. This BMP9 trend in LPS-stimulated hepatic stellate cells mirrored the findings observed in vivo. Mechanistically, the involvement of H3K9 dimethylation and G9a methyltransferase in this process was elucidated. Treatment with UNC0642 successfully restored suppressed BMP9 levels. Chromatin immunoprecipitation assays further confirmed an increased enrichment of H3K9 dimethylation in the promoter region of the BMP9 gene following LPS stimulation of mouse hepatic stellate cells.

**Conclusion:**

These findings suggested that LPS in pulpitis might transiently influence the expression of BMP9 in liver tissues, and pulpitis might have potential effects on systemic tissues and organs.

**Clinical relevance:**

This study provides a proof-of-concept for targeting epigenetic pathways to mitigate the oral-systemic disease link, motivating future clinical trials in high-risk populations.

## Introduction

The connections between localized oral infectious diseases and systemic organs are well-documented. Apical periodontitis has been established to have direct associations with various systemic diseases, including type 2 diabetes, cardiovascular diseases, and immune-mediated disorders.^1^, ^2^, ^3^, ^4^ Recent studies have indicated that diabetes could impact the healing of pulp and apical tissues.^5^ Furthermore, it has been confirmed that the incidence of pulp stones is significantly higher in patients with heart-related diseases.^6^^,^^7^ This suggests that chronic oral infections may serve as a persistent source of inflammation, potentially representing a shared pathological mechanism influencing overall systemic health. Pulpitis, a common and frequently occurring oral disease, is an infectious disease, and its potential impact on the systemic system deserves further investigation.

Most pulpitis cases are caused by oral bacterial infection, with large numbers of bacteria and their metabolites present at inflammatory sites.^8^^,^^9^ In severe infections, such as infectious intestinal diseases, it is recognized that bacteria and their metabolites could infiltrate the gut-derived lymph or bloodstream via compromised vascular endothelium, consequently inciting pathological manifestations in distant tissues or organs.^10^ Similarly, bacteria in periodontitis can affect other tissues and organs through blood circulation or the gastrointestinal tract.^11^ However, conclusive evidence is lacking regarding whether bacteria or their metabolites from pulpitis could reach other parts of the body.

Bone morphogenetic protein 9 (BMP9), a vital component of the BMP family, exerts a pivotal role in the regulation of metabolism and inflammation, and serves as a fundamental regulatory gene in liver fibrosis.^12^, ^13^, ^14^, ^15^ Recently, BMP9 is increasingly recognized as a pleiotropic homeostatic regulator that plays critical roles in diverse physiological and pathological processes. In the liver, BMP9 helps maintain sinusoidal endothelial cell quiescence, though dysregulated signalling may also promote fibrosis. Metabolically, BMP9 combats obesity and related disorders by modulating glucose metabolism and insulin signalling pathways.^16^ In the cardiovascular system, elevated BMP9 levels are observed in patients with heart failure and myocardial infarction, and experimental models demonstrate its protective role against cardiac fibrosis through suppression of TGF-β1 signalling and collagen synthesis.^17^^,^^18^ Together, these findings highlight BMP9 as a promising transorgan, transdisease regulatory molecule and support its potential as a therapeutic target.

Unlike other BMP family members that have been extensively studied, BMP9 is primarily synthesized in the liver and exerts both autocrine and paracrine effects.^19^ Notably, BMP9 can enter the bloodstream and remain in a bioactive state without undergoing degradation, allowing it to participate in maintaining vascular homeostasis.^20^, ^21^, ^22^ Additionally, intraperitoneal administration of lipopolysaccharide (LPS) has been shown to impact the expression of BMP9 in the liver.^15^

Our previous study demonstrated that BMP9 potently promoted the odontogenic differentiation of dental pulp cells and pulp injury repair. We also observed a dynamic pattern of BMP9 expression in pulpitis. This pattern was characterized by an initial decrease, followed by a subsequent increase, aligning with earlier reports on the impact of LPS on liver BMP9 expression. Notably, the elevated BMP9 expression was more pronounced in the vicinity of blood vessels.^23^^,^^24^ Given that BMP9 exists in blood vessels in a biologically active state, it was hypothesized that BMP9 in pulpitis might be partly derived from blood vessels. As BMP9 in blood vessels originates from liver tissue, it was further speculated whether BMP9 could serve as a bridge connecting pulpitis and liver tissue.

Based on the findings presented, we hypothesize that pulpitis may dynamically regulate hepatic BMP9 expression through LPS-mediated epigenetic reprogramming in hepatic stellate cells, thereby establishing a novel potential mechanism through which local oral infection influences systemic metabolic homeostasis.

## Materials and methods

All animal studies were approved by the Ethical Committees of Stomatology and the State Key Laboratory of Oral Diseases (WCHSIRB-D-2020-368).

### Establishment of pulpitis in rats and mice

To verify whether pulpitis could affect the expression of BMP9 in the liver, a rat pulpitis model was constructed.^24^ Twenty Sprague-Dawley rats (male, 8 weeks old, approximately 220 g in weight) were randomly divided into four groups based on the sample collection timepoint after pulp chamber opening: day 1 (D1), day 3 (D3), day 7 (D7), and NS (control group, without treatment). The animals were subjected to general intraperitoneal anaesthesia (sodium pentobarbital). For each animal, a cavity was made on the occlusal face of the mandibular first molars using a spherical burr at high speed without cooling. Rats were sacrificed at different time points, and liver tissues were immediately collected and fixed in paraformaldehyde (Biosharp) for 24 hours.

**Fig. 1:**
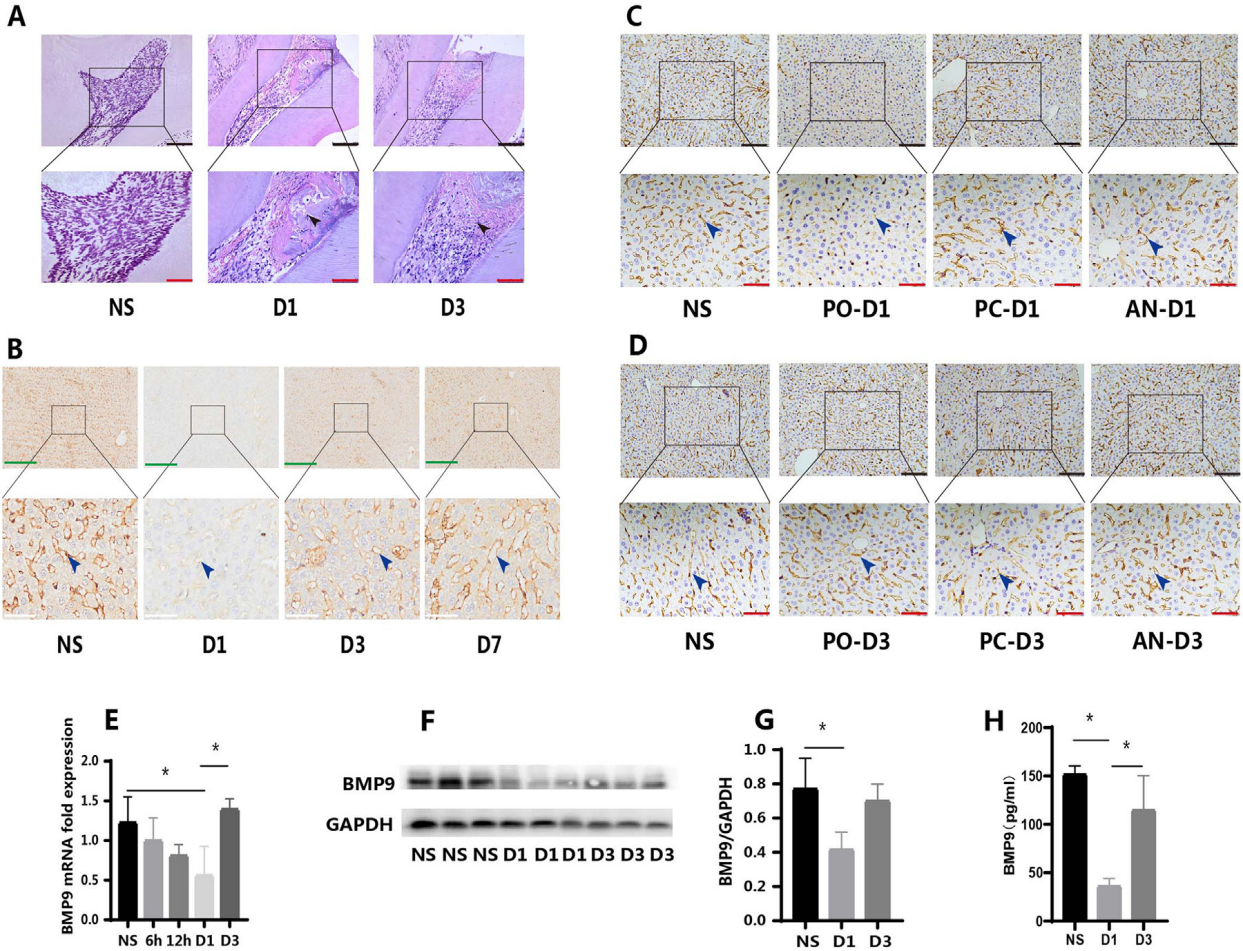
Changes of BMP9 expression in liver after pulp opening in rats/mice. (A) Haematoxylin–eosin (HE) staining of mice pulpitis tissues (black arrows: inflammatory cell). (B) Expression of BMP9 in rat liver occurred mainly in the peripheral cells of the sinusoid (blue arrows); it decreased significantly at D1 and returned to normal at D3 and D7 (IHC). (C) At D1 after pulp opening, the expression of BMP9 in mice liver of the PO group decreased significantly, but there was no significant change in the PC or AN groups (blue arrows) (IHC). (D) At D3 after pulp opening, the expression of BMP9 in liver tissue of mice in PO group increased, returning to the normal level, whereas there was no significant change in other groups (IHC). (E) RT-qPCR results showed that BMP9 gene expression began to decline at 6 hours after pulp opening and reached its lowest level at D1, before returning to normal levels. (F and G) WB results showed that the expression of BMP9 protein decreased at D1 after pulp opening and increased at D3. (H) ELISA showed that BMP9 levels in the blood of mice decreased at D1 and rose at D3. AN, anaesthetic; D, day; NS, normal sample; PC, pulp capping; PO, pulp opening. Data are presented as mean ± SD; *n* = 6; *P < .05. Black scale: 100 μm, red scale: 50 μm; green scale: 150 μm; white scale: 25 μm.

Following the experimental results in rats, further verification of the results’ accuracy was conducted in C57BL/6J (C57) mice. To exclude the effects of intraperitoneal injection of anaesthetic and confirm that its possible effect was related to oral microbial infection, an anaesthetic group and pulp capping group were established. The anaesthetic group received intraperitoneal injection of anaesthetic without other treatment. The experiment included a normal group, pulp opening group, anaesthetic group, and pulp capping group at each time point (Figure 1A), with six mice (male, 8 weeks old, approximately 22 g in weight) in each group (no treatment was given at the 0 hour time point, and a total of six mice were used as controls). Liver tissue and blood of mice were collected at 6, 12, 24, and 72 hours after pulp opening for follow-up tests. The pulp opening procedure in mice’s teeth was similar to that in rats but was performed under a stereo microscope (Leica). Using the stereo microscope, the pulp of the maxillary first molars of mice was opened by minimally invasive drilling. Liver tissue and blood were collected at different time points. The liver tissue was immediately fixed with paraformaldehyde for 24 hours. Blood samples were allowed to stand at room temperature for 1 hour, then centrifuged at 3000 rpm and 4°C for 15 minutes, and the supernatant was collected.

### Cell culture

For the culture of mouse hepatic stellate cells (mHSCs), an improved version of the previously reported culturing method was employed, including liver perfusion, liver cell removal, cell gradient centrifugation, morphology, and marker verification.^25^^,^^26^ Initially, mice were anesthetized via intraperitoneal injection, and the abdominal skin was disinfected. After exposing the liver area, the hepatic portal vein was infused with Hanks solution without calcium or magnesium (Solarbio) until the liver turned yellow. Subsequently, the liver tissue was excised and placed in an appropriate amount of type IV collagenase (2 mg/mL, Solarbio), homogenized in a sterile cell culture dish, and gently incubated at 37°C for 25 minutes.

The digested liver tissue fluid was filtered through a 100-μm cell strainer (Biologix). Centrifugation was performed at 1700 rpm and 4°C for 6 minutes, after which the supernatant was removed. Hanks solution was used to resuspend the cells, followed by centrifugation at 400 rpm and 4°C for 5 minutes, and the supernatant was collected. A second centrifugation was performed at 1700 rpm and 4°C for 6 minutes, and the supernatant was discarded.

A physiological-concentration Percoll (Signalway Antibody) (100%) solution was prepared with 8.5% sodium chloride solution, and a 60% Percoll solution was prepared with 0.85% sodium chloride solution. The precipitate obtained in the previous step was mixed with 2 mL of Percoll at physiological concentration and slowly added along the centrifuge wall to the 60% Percoll solution. The mixture was centrifuged at 5000 rpm and 4°C for 21 minutes, and the surface sediment was transferred to a new centrifuge tube. Hanks solution was added once more, followed by centrifugation at 1700 rpm and 4°C for 6 minutes; this step was repeated. The supernatant was removed, and the precipitate was resuspended in DMEM (Gibco) supplemented with 20% foetal bovine serum (Gibco), 50 mg/mL streptomycin, and 100 U/mL penicillin (Sigma-Aldrich) before being transferred to a 10-cm dish for culture. The culture medium was changed after 48 hours. After the cells reached 90% confluence, they were subcultured for subsequent tests.

### Immunohistochemistry (IHC)

The tissue was embedded in paraffin and cut into 5-μm sections. Paraffin sections were dewaxed in xylene, rehydrated with distilled water, and then subjected to antigen retrieval using sodium citrate (Solarbio) for 15 minutes at 95°C or proteinase K (Solarbio) for 15 minutes at 37°C. Standardized IHC kits were used (Zhongshanjinqiao). The slides were incubated overnight at 4°C with antibodies against BMP9 (1:500, sc-514211, Santa Cruz Biotechnology) and LPS (1:100, KL20041-01, KangLang Biological).

### Immunofluorescence (IF)

Liver tissue after perfusion was collected for IF staining. The process of tissue fixation, embedding, dewaxing, and antigen repair was the same as that used for IHC. The slides were sealed with 5% bovine serum albumin (BSA) at room temperature for 1 hour; then, primary antibodies against BMP9 (Santa Cruz Biotechnology) and desmin (1:100, 48,749, Signalway Antibody) from different species were added, followed by incubation overnight at 4°C. Secondary antibodies (FITC, Zhongshanjinqiao; Cy3, Solarbio) from different species were added with incubation at room temperature for 1 hour. Appropriate amounts of DAPI solution were added, followed by incubation for further 3 minutes at room temperature. Finally, images were obtained under a fluorescence microscope (Leica).

For immunocytofluorescent, cells of different treatment groups in 48-well plates were fixed with paraformaldehyde, further permeabilized with Triton X-100, blocked with 5% BSA, and incubated overnight with primary antibodies against dimethylation of lysine 9 of histone 3 (H3K9me2; Signalway Antibody) and BMP9 (Santa Cruz Biotechnology). Cells were then incubated with the fluorescent secondary antibody (HUABIO) for 1 hour, followed by addition of DAPI and further incubation. Fluorescence microscopy was performed to obtain images.

### RNA isolation and real-time quantitative PCR (RT-qPCR)

The liver tissue was placed into a grinding EP tube and homogenized with an appropriate amount of TRIzol reagent and grinding beads using a grinding instrument (Servicebio). Subsequently, the RNA extraction process followed the same protocol as that used for cell extraction. Total RNA from six-well plate cells was isolated using TRIzol reagent (Invitrogen) according to the manufacturer’s protocol. The extracted RNA products were then reverse transcribed and quantitatively analysed. Glyceraldehyde-3-phosphate dehydrogenase (GAPDH) was used as an internal control. Primers were designed to generate products of approximately 200 base pairs for efficient analysis. Primer sequences are provided in the appendix materials (Supplementary Tables 1 and 2).

### Western blot (WB) analysis

Liver tissue was placed in an appropriate amount of total protein lysate (Signalway Antibody) and homogenized to extract total protein, following a process similar to RNA extraction. The subsequent protein extraction process was identical to that used for cell extraction from 6-cm dishes. Protein concentration was measured using a BCA protein assay kit (Thermo Scientific). Denatured protein samples (20-40 μg) were loaded onto a 10% polyacrylamide gel and separated by electrophoresis. Proteins were transferred to a polyvinylidene fluoride membrane (Millipore), and 5% skimmed milk powder or BSA (Solarbio) was used for antigen blocking. Samples were then incubated overnight with primary antibodies including anti-BMP9 (1:500, Santa Cruz Biotechnology), anti-H3K9me1/2/3 (HW004/HW005/HW029, 1:1000, Signalway Antibody), H3K27 me1/2/3 (HW006/HW007/HW008, 1:1000, Signalway Antibody), Ezh2/GAPDH (33,009/21,612, 1:1000/1:50,000, Signalway Antibody), G9a (YN2286, 1:1000, ImmunoWay), and SETDB1/SUV39H (EM1709-76/ET7108-37, 1:1000 HUABIO). Secondary antibodies were antimouse/rabbit immunoglobulin G conjugated to horseradish peroxidase (sc-2005/sc-2004, 1:10,000; Santa Cruz Biotechnology). Immunoblot bands were visualized by enhanced chemiluminescence using a Bio-Rad detection system (Bio-Rad).

### Enzyme-linked immunosorbent assay (ELISA)

LPS (Signalway Antibody) and BMP9 (Abcam) in mouse blood were detected according to the corresponding ELISA instructions using the following general process. All ELISA reagents were passively restored to room temperature, and then standard samples and control samples were added to the well plate for incubation. The well plate was then cleaned with a cleaning solution (300 μL per hole). Follow-up reagents were added successively according to the standard procedure. Absorbance was measured at 450 nm, and the corresponding concentration was calculated.

### UNC0642 inhibitor experiment

For in vitro experiments, mHSCs were cultured into 6-cm dishes or 24-well plates and divided into a dimethyl sulfoxide (DMSO) control group; DMSO + LPS group; and 0.5, 1, and 5 μM UNC0642 (Selleck) groups. After the cells reached 90% confluence, different molar concentrations of inhibitors were added. After 24 hours of treatment, *Porphyromonas gingivalis* (*P.g*) LPS (Invivogen) was added to cells in the DMSO + LPS group and inhibitor groups. Samples were collected at 4 hours for WB or immunocytofluorescent tests.

In vivo tests were performed with UNC0642 (5 mg/kg). The working solution formula was as follows: 5% DMSO + 40% PEG300 + 5% Tween80 + double-distilled H2O, with a final concentration of 7.5 mg/mL. Eighteen C57 mice (male, 8 weeks old, about 22 g in weight) were divided into three groups, namely DMSO (control group, DMSO stands for the in vivo working fluid of UNC0642), DMSO-PO (PO: pulp opening), and UNC0642-PO groups. The corresponding groups were given intraperitoneal injections of working solution and UNC0642 inhibitors on the first day. The next day, a pulp opening test was performed on the maxillary first molars of mice. The inhibitor was injected again at 24 hours after the first intraperitoneal injection to ensure sufficient blood concentration. The mouse liver samples were collected at 24 hours after pulp opening.

### Chromatin immunoprecipitation (ChIP) analysis

mHSCs were seeded in six 10-cm cell culture dishes and divided into NS (control group, without treatment) and LPS groups. Upon reaching 90% confluence, *P.g* LPS was added to the LPS group. After 4 hours of incubation, cells were harvested for ChIP assay using an Abcam ChIP kit (ab500, Abcam) according to the manufacturer’s instructions. Cells were fixed with 37% formaldehyde at a final concentration of 1%, and the reaction was terminated with 125 mM glycine. Digested cells were subjected to sonication treatment, followed by immunoprecipitation. Chromatin was precipitated using rabbit anti-H3K9me2 and IgG (negative control) antibodies. Immunoprecipitated DNA was amplified via RT-qPCR using primers corresponding to the BMP9 promoter region. The final data are presented as percentages of input DNA.

## CCK8 assay

According to the CCK8 (Beyotime) assay instructions, it is roughly as follows. The mHSCs were inoculated into 96-well plates. When the cells were fused to 90%, *P.g* LPS was added, and the concentrations were 1, 2.5, 5 μg/mL, and NS was used as the control group. An amount of 10 μL CCK8 was added into each well 48 hours later. After further incubation for 4 hours, the absorbance value was measured at 450 nm.

## Statistical analysis

Data from at least three independent experiments were given as mean ± SD. The Shapiro–Wilk normality test was used to determine the normality of the sample distribution (*P* > .05). Student’s *t* test was used to test the significance of differences between two independent samples. One-way analysis of variance followed by Tukey’s post hoc test was used for multiple comparisons. Statistical significance was defined as two-tailed *P* < .05.

## Results

### Expression of BMP9 in rats/mice after pulpitis

IHC results in rat liver tissues revealed that BMP9 was predominantly expressed in perivascular cells (Figure 1B, blue arrow). A significant decrease in BMP9 expression was observed on D1 (Figure 1B, D1), with notably lighter staining intensity. However, BMP9 expression was upregulated on D3 and D7, reaching levels comparable to those of the normal group (Figure 1B, D3 and D7).

In mice, BMP9 was similarly found to be primarily expressed in hepatic perivascular cells (Figure 1C, D, blue arrow). In the pulp opening group, BMP9 expression decreased at 24 hours (Figure 1C, PO-D1) but recovered to levels consistent with the normal group at 72 hours (Figure 1D, PO-D3). Furthermore, BMP9 expression levels in the normal, anaesthetic, and pulp capping groups were comparable in terms of staining area and intensity at different time points (Figure 1C, D).

RT-qPCR results demonstrated that BMP9 mRNA expression began to decrease at 6 hours, although this decrease was not statistically significant. A decreasing trend in BMP9 mRNA expression was observed at 12 hours, with the most pronounced decrease at 24 hours. Expression levels recovered at 72 hours, consistent with the normal group (Figure 1E). WB analysis revealed decreased BMP9 protein expression at 24 hours, while expression levels at other time points were similar to those of the normal group (Figure 1F, G). The observed temporal difference between gene and protein expression changes was logically consistent.

Given that BMP9 existed in the blood in a bioactive state, changes in BMP9 levels in the blood were also examined. ELISA results showed decreased BMP9 levels in blood at 24 hours and increased levels at 72 hours, consistent with the observations in liver tissue (Figure 1H).

### Changes of LPS concentrations in blood and liver tissues of mice

**Fig. 2:**
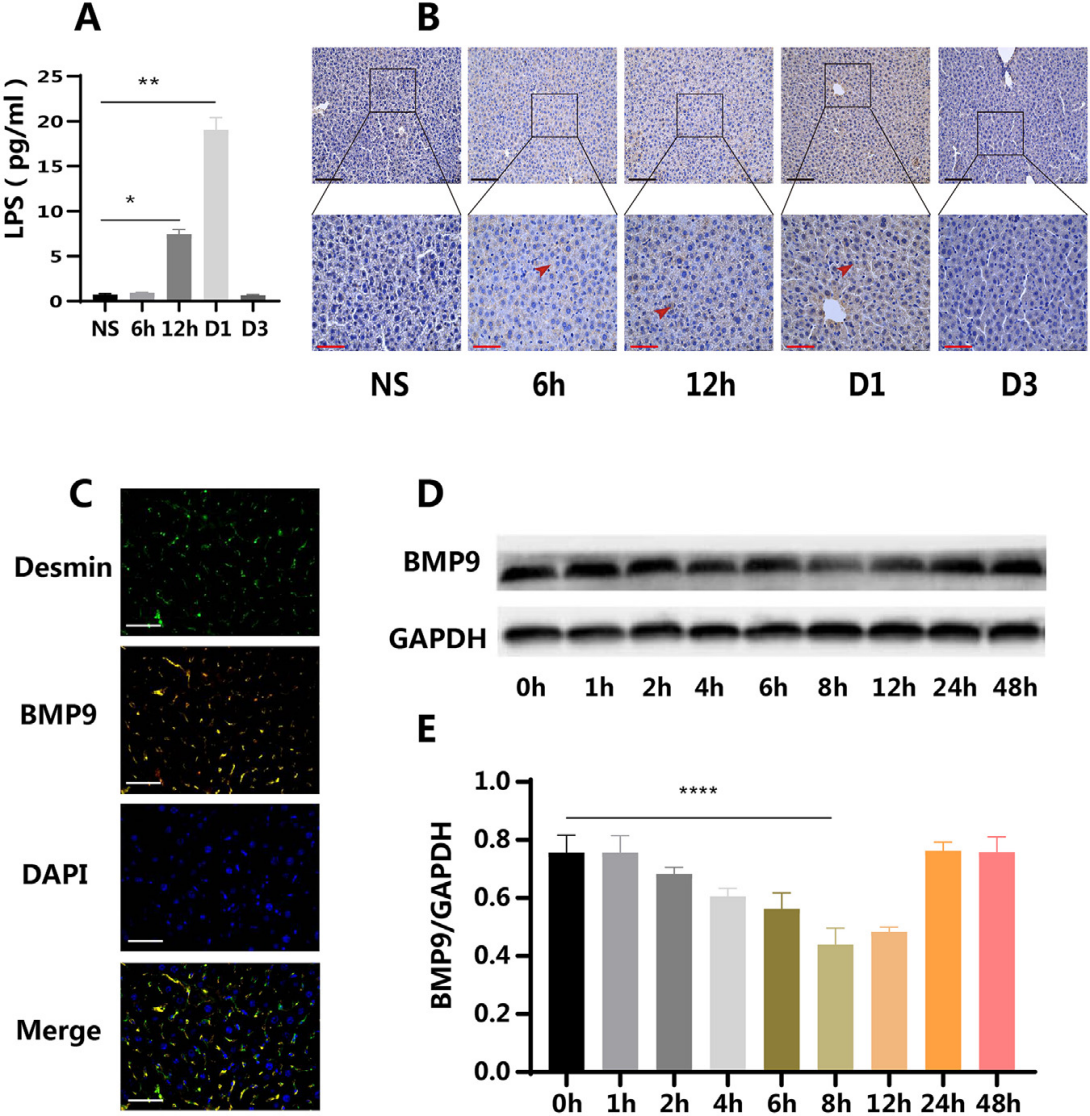
Changes of LPS concentration in liver and blood of mice after pulp opening and BMP9 expression in mHSCs stimulated by *P.g* LPS. The concentration of LPS in the blood (A) and liver (B) began to rise at 12 hours, peaking at D1 and dropping to basal level at D3 (ELISA and IHC) (*n* = 6). (C) The expression sites of BMP9 (yellow) and demsin (green) overlapped in liver tissue (IF) (*n* = 3). (D and E) *P.g* LPS inhibited the expression of BMP9 in mHSCs cultured in vitro for a short time (WB) (*n* = 3). D, day; NS, normal sample. Data are presented as mean ± SD; *P < .05; **P < .01. Black scale: 100 μm, red scale: 50 μm; white scale: 50 μm.

After establishing a relationship between pulpitis and liver BMP9 expression, further investigation was conducted to elucidate the underlying mechanism of this interaction. Initially, changes in LPS levels in the blood of mice were examined. ELISA results revealed that blood LPS concentration began to increase 6 hours after tooth pulp opening, reaching its peak at 24 hours, and returning to baseline levels at 72 hours (Figure 2A). Furthermore, IHC staining was performed on liver tissue to detect changes in LPS concentration. The results demonstrated that liver LPS levels increased at 6 hours postpulp opening, remained elevated until 24 hours, and returned to normal levels at 72 hours, consistent with the ELISA findings (Figure 2B). These observations suggest a temporal correlation between pulpitis-induced systemic LPS levels and the alterations in liver BMP9 expression, providing a potential mechanistic link between oral inflammation and hepatic responses.

### BMP9 expression was downregulated in *P.g* LPS-stimulated mHSCs

Subsequent investigations focused on identifying the source cells of liver BMP9. Fluorescence-based visualization revealed colocalization of the HSC marker desmin and BMP9 (Figure 2C). In conjunction with the IHC results, this observation suggested that mHSCs were the primary source of BMP9 in the liver. In vitro culture of mHSCs was then performed. Under optical microscopy, the cells were observed to transition from a round to an irregular shape during culture, developing star-shaped or polygonal protrusions consistent with the growth pattern of HSCs after adherence (Supplementary Figure 1A, B). Fluorescence microscopy confirmed that these morphologically altered cells expressed desmin and α-SMA proteins (Supplementary Figure 1C, D). For the first time, the optimal concentration of *P.g* LPS for stimulation of mHSCs was determined. Based on the expression levels of IL-6 and BMP9, the optimal *P.g* LPS concentration was established at 2.5 μg/mL (Supplementary Figure 2). WB analysis demonstrated that following *P.g* LPS stimulation of mHSCs, BMP9 protein expression decreased significantly at 8 hours and gradually recovered by 12 hours (Figure 2D, E), mirroring the pattern observed in vivo.

These findings provided an insight into the cellular source of BMP9 in the liver and established a model for investigating the effects of bacterial products associated with pulpitis on hepatic BMP9 expression.

### Changes in H3K9 and H3K27 methylation and related methyltransferase expression

**Fig. 3:**
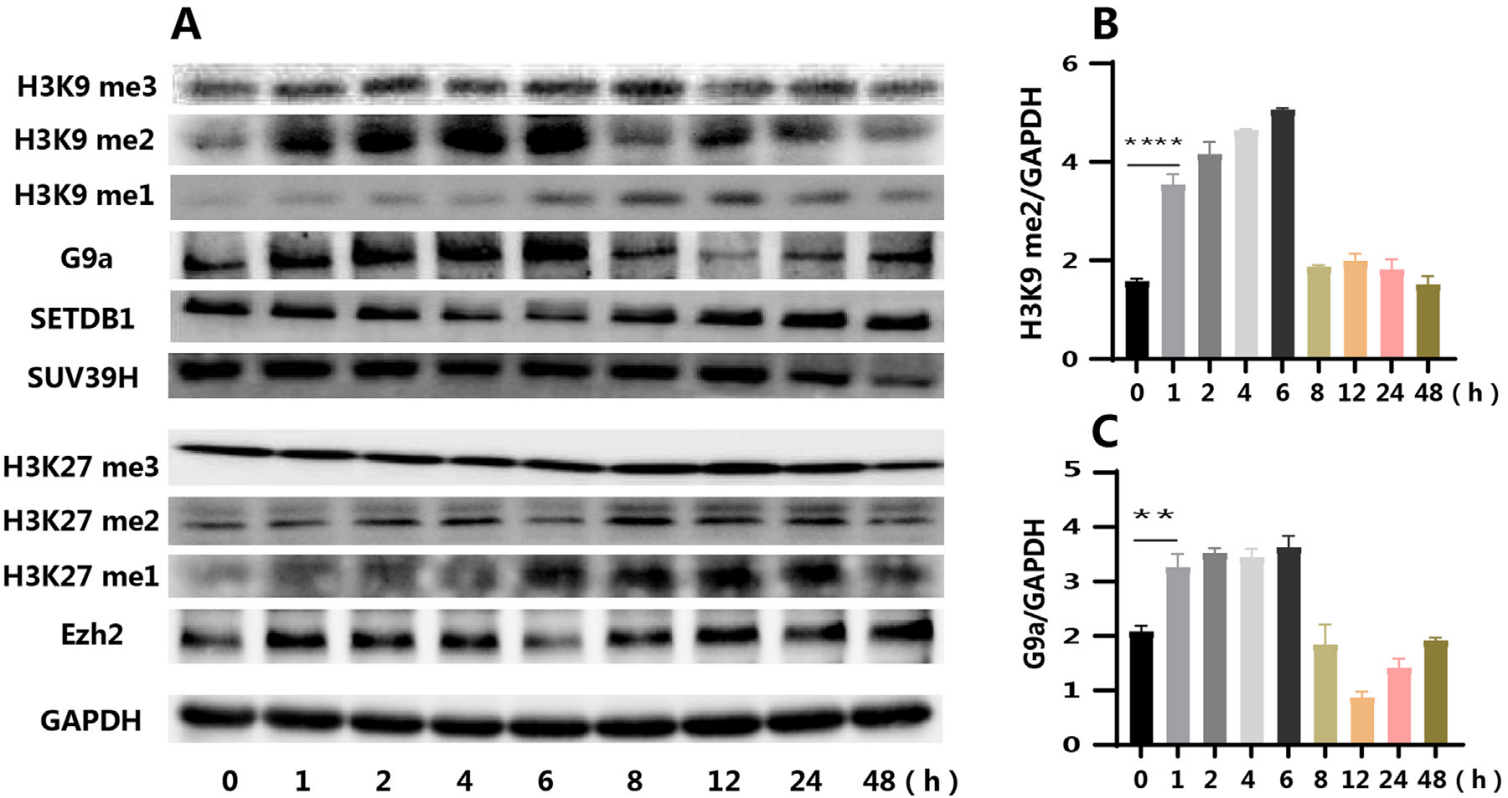
Changes in methylation levels of H3K9 and H3K27 and expression of related methyltransferases in mHSCs stimulated by *P.g* LPS. (A) The different methylation levels of H3K9 and H3K27 and expression of H3K9 and H3K27 methyltransferases G9a, SETDB1, SUV39H, and Ezh2 (WB). (B) The change in H3K9me2 levels. (C) The change in G9a expression. me1/2/3 stands for monomethylation, dimethylation, and trimethylation, respectively. Data are presented as mean ± SD; *n* = 3; **P < .01.

Levels of histone methylation at H3K9 and H3K27, which commonly causing gene silencing, were subsequently measured. Following *P.g* LPS stimulation of mHSCs, both H3K9 and H3K27 methylation levels changed. However, the alterations in H3K27 methylation were not substantial and did not align with the trends observed for BMP9 expression (Figure 3A). H3K9me2 levels increased from 1 to 6 hours, consistent with the previously observed trend of decreased BMP9 expression (Figure 3A, B). Changes in the expression of methyltransferases related to H3K9 and H3K27 methylation were then examined. The results revealed that the expression of H3K9 methyltransferase SUV39h2 decreased gradually with increasing stimulation time, while SETD1 expression initially decreased and then increased at 8 hours (Figure 3A). The expression of G9a methyltransferase began to increase at 1 hour, reached its peak at 6 hours, and subsequently decreased, mirroring the changes observed in H3K9me2 levels (Figure 3A, B). Based on these observations, G9a was selected as the key molecule for subsequent investigation, given its temporal correlation with H3K9me2 levels and BMP9 expression changes.

### UNC0642 inhibited downregulation of BMP9 in vitro and in vivo

**Fig. 4:**
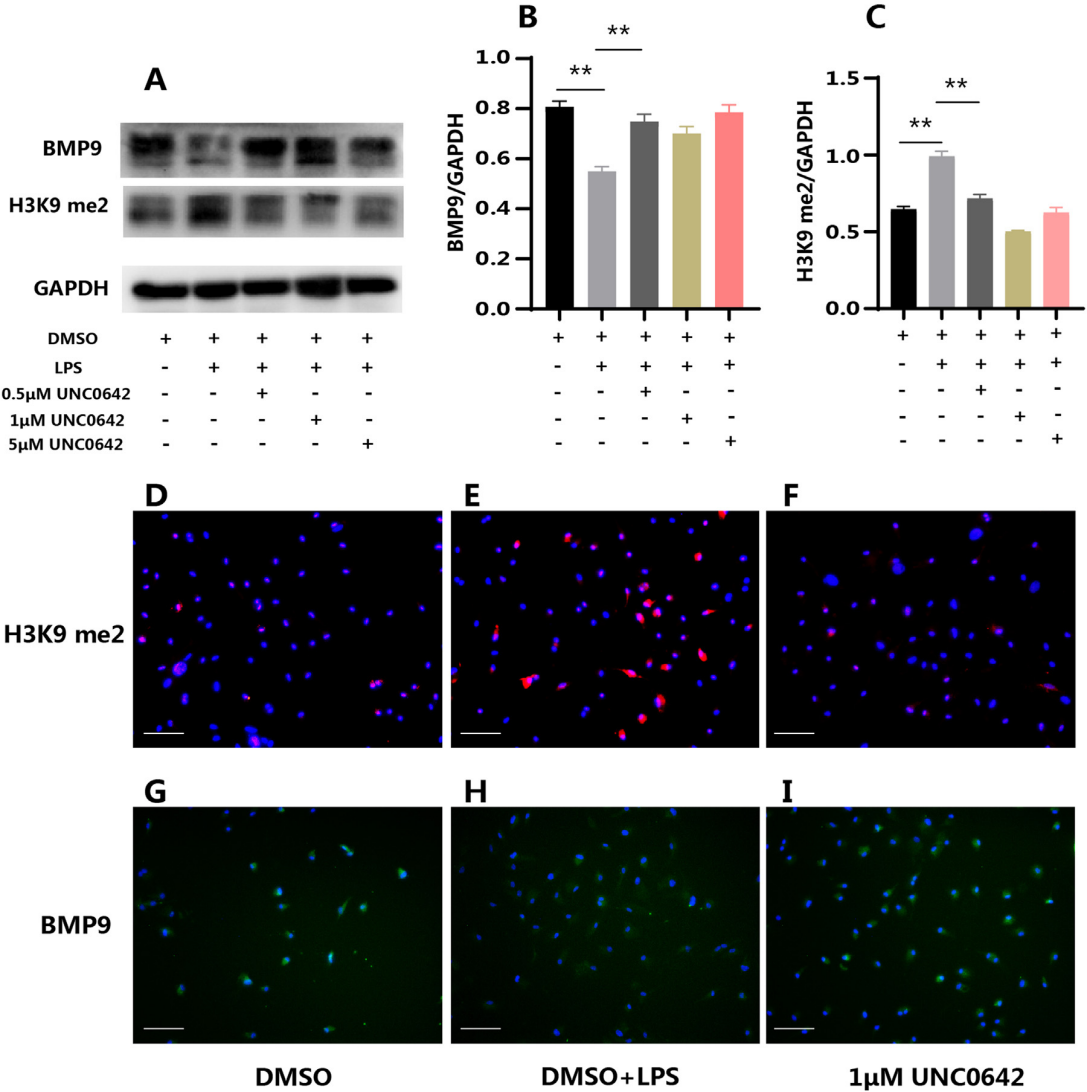
UNC0642 inhibited the downregulation of BMP9 expression in *P.g* LPS-stimulated mHSCs in vitro. (A) *P.g* LPS inhibited the expression of BMP9 and increased the H3K9me2 levels. UNC0642 at different molar concentrations restored the expression of BMP9 and inhibited H3K9me2 levels (WB). (B) The changes in BMP9 expression. (C) The changes in H3K9me2 levels. (D-I) *P.g* LPS reduced the fluorescence intensity of BMP9 and increased that of H3K9me2. 1 μM UNC0642 restored the fluorescence intensity of BMP9 and inhibited that of H3K9me2 (ICF). me2: dimethylation. Data are presented as mean ± SD; *n* = 3; **P < .01.

UNC0642, a specific inhibitor of G9a, was then used to pretreat the cells. WB results showed that *P.g* LPS stimulation upregulated H3K9me2 levels, whereas UNC0642 inhibited H3K9me2 levels (Figure 4A, C) and, most importantly, restored BMP9 expression (Figure 4A, B). IFC further confirmed the same results (Figure 4D-I).

**Fig. 5:**
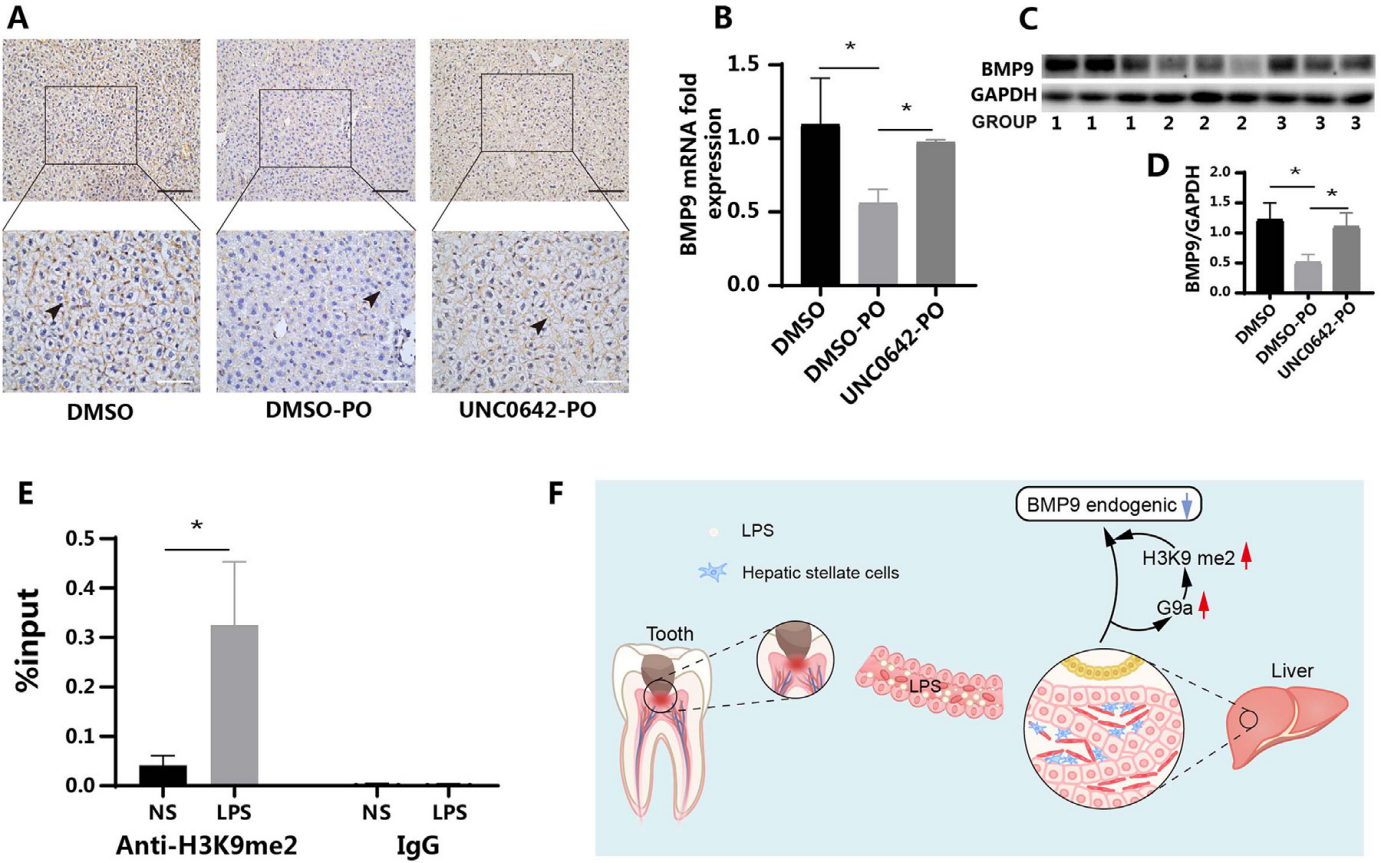
UNC0642 restored BMP9 expression in C57 mice and binding of H3K9me2 to the promoter region of BMP9 gene. (A) The expression of BMP9 in the DMSO-PO group was decreased, whereas that in the UNC0642-PO group was upregulated and similar to that in the DMSO control group (WB). (B) The RT-qPCR results are consistent with the IHC results. (C and D) The WB results were consistent with the IHC results (*n* = 6). The groups 1 to 3 correspond to the DMSO group, DMSO-PO group, and UNC0642-PO group, respectively. (E) After stimulation of mHSCs with *P.g* LPS, the enrichment rate of H3K9me2 in the promoter region of BMP9 gene was significantly increased (ChIP) (*n* = 3). (F) Full-text pattern diagram. LPS from pulpitis reached the liver through the bloodstream, stimulating hepatic stellate cells and regulating the expression of BMP9 in the liver via H3K9 dimethylation. DMSO represents the working fluid of inhibitors in vivo: DMSO + 40% PEG300 + 5% Tween80 + double-distilled H2O; LPS, samples stimulated with *P.g* LPS; me2, dimethylation; NS, normal sample; PO, pulp opening. Data are presented as mean ± SD; *P < .05. Black scale: 100 μm, white scale: 50 μm.

Then, we verified the function of G9a in mice using IHC. The staining degree of BMP9 in the DMSO and UNC0642-PO groups showed no significant change, but there was a decrease in the DMSO-PO group (Figure 5A). Liver tissue was extracted from mice for further examination. RT-qPCR and WB results showed that compared to the DMSO group, the mRNA and protein expression of BMP9 decreased in the DMSO-PO group, whereas the UNC0642-PO group showed no significant change, consistent with the IHC results (Figure 5B-D).

### Binding of H3K9me2 to the BMP9 promoter region was enhanced

The aforementioned results confirmed an association between decreased BMP9 expression and increased H3K9me2 levels in the BMP9 promoter region. The ChIP results demonstrated that *P.g* LPS stimulation significantly enhanced the enrichment of H3K9me2 in the promoter region of the BMP9 gene in mHSCs, suggesting the regulation of BMP9 expression by H3K9me2 (Figure 5E). The major steps involved in this research study have been summarized in a PRILE 2021 flowchart, as illustrated in Figure 5F This concise summary emphasized the key experimental findings and provides a visual representation of the study’s workflow, enhancing the clarity and accessibility of the research.

## Discussion

The interconnections between oral and systemic diseases are extensive; however, the relationship between pulp diseases and other body systems has been insufficiently explored. The limited studies to date have primarily focused on the association between pulp stones and ischemic heart disease, or the influence of hyperglycaemia on pulp injury healing.^6^^,^^27^ Given that pulpitis is an infectious disease and the pulp tissue is highly vascularized, its potential systemic effects warrant investigation.

The current study revealed that, in both rat and mouse models of pulpitis, hepatic BMP9 expression was inhibited on the first day following inflammation, subsequently returning to baseline levels. Interestingly, this phenomenon was not observed in the mouse pulp capping group, implying that microorganisms or their products at the pulp inflammation site might be the primary contributors. Previous research has indicated that intraperitoneal administration of LPS results in a transient decrease in hepatic BMP9 expression in mice, followed by a subsequent increase.^15^ Elevated levels of bacterial metabolites have been detected in the serum of patients with periodontal or periapical diseases. These harmful substances, upon reaching other parts of the body, could cause damage and activate immune responses.^11^^,^^28^ Given that blood circulation served as a plausible connection between pulpitis and the liver, it was suggested that bacterial metabolites from pulpitis entering the bloodstream might exert effects on hepatic tissue.

The results showed that pulpitis increased serum LPS levels, with corresponding changes in the liver. Importantly, a significant negative correlation was found between LPS concentration and BMP9 expression, hinting at a potential causal link. The subsequent drop in LPS levels may be due to the body’s immune system clearing harmful substances as BMP9 expression returns to normal.

This study provided a novel insight into the systemic consequences of pulpitis, especially its influence on hepatic BMP9 expression, and underscores the potential role of bacterial metabolites in this interaction. HSCs, situated in the pericentral space of the hepatic sinusoid near sinusoid endothelial cells and hepatocytes, are vital liver parenchymal cells. As the main cells secreting the extracellular matrix in liver tissue, HSCs are closely linked to liver fibrosis.^29^, ^30^, ^31^ BMP9 is also recognized as a crucial regulator of liver fibrosis.^19^^,^^20^ Most studies agreed that mHSCs are the source of BMP9 in the liver, and our IHC and IF results supported this conclusion.^15^^,^^19^^,^^32^

In vitro stimulation of mHSCs with *P.g* LPS yielded similar changes in BMP9 expression to those observed in vivo, albeit with an earlier decline in BMP9 expression. Previous research using *Escherichia coli* LPS for in vitro stimulation of mHSCs reported decreased BMP9 expression at 24 hours, whereas the current study found that BMP9 expression levels had already been restored by this time point.^15^ This discrepancy may be attributed to differences in LPS toxicities; however, the overall trend in BMP9 expression changes remained consistent. These findings suggested that bacterial infections may influence hepatic BMP9 expression. Moreover, it is essential to explore the potential amplification of this effect on liver BMP9 expression when dental pulp infection coincides with pathological reactions in hepatic tissue. These inquiries hold immense value in elucidating the intricate interplay between dental pulp infection and the modulation of BMP9 expression in liver tissue.

The investigation proceeded to elucidate the mechanism by which LPS regulates BMP9 expression. Epigenetic regulation has been established as a means of modulating gene expression without altering gene sequences.^33^^,^^34^ Histone modification represents a crucial form of epigenetic regulation, with methylation of histone H3K9 and H3K27 associated with transcriptional repression.^35^^,^^36^ Epigenetic mechanisms regulate numerous genes functioning in HSCs, and external stimuli such as alcohol and hypoxia can induce changes in epigenetic regulation within these cells.^37^ LPS has been shown to upregulate IL-8 expression, accompanied by transient fluctuations in H3K9 methylation levels, initially exhibiting a brief increase followed by a subsequent decline.^38^ Conversely, H3K9 methylation and G9a methyltransferase have been implicated in regulating TNF-α gene silencing in the LPS-tolerant phenotype observed in leukocytes.^39^^,^^40^

The present study showed that H3K9me2 and G9a have a key role in the mechanism by which *P.g* LPS regulates BMP9 expression. G9a has been confirmed to be the methyltransferase of H3K9 monomethylation and dimethylation, and UNC0642 is a competitive inhibitor of G9a and GLP (G9A-like protein) substrates, with excellent in vitro and in vivo inhibitory activity and low cytotoxicity.^41^^,^^42^ Both in vitro and in vivo results demonstrated that UNC0642 restored BMP9 expression following inhibition by *P.g* LPS, confirming G9a’s regulatory role in this process. The ChIP results in this study confirmed that H3K9me2 regulates BMP9 gene expression following *P.g* LPS stimulation. These findings elucidated the underlying mechanism through which dental pulp infection influenced BMP9 expression in liver tissue and provide valuable insights into the involvement of epigenetic mechanisms in the complex interplay between oral infectious diseases and other systemic tissues and organs.

Our previous research demonstrated a distinct pattern of BMP9 expression at sites of pulp inflammation, characterized by an initial decrease followed by subsequent upregulation, with elevated expression predominantly observed in perivascular regions.^24^ Consistent with these findings, the present study revealed a similar trend of initial decline and subsequent upregulation in both hepatic tissue and circulating BMP9 levels following pulpitis induction. The results of this study supported the aforementioned hypothesis. This finding provided direct experimental evidence and a mechanistic explanation for the hypothesis that pulpitis may act as a risk factor for systemic diseases, in part by disrupting the expression of the hepatic homeostatic factor BMP9. This study reveals that pulpitis may regulate hepatic BMP9 expression through the LPS-G9a-H3K9me2 axis, providing a novel mechanism for understanding how oral infections influence systemic homeostasis. Recent studies have reported that HDAC9-mediated histone deacetylation promotes osteoclast differentiation and disease progression by inducing mitochondrial dysfunction and pyroptosis in periodontal ligament fibroblasts.^43^ This finding further reveals the central role of epigenetic regulation in how oral diseases affect local tissue homeostasis. Clinically, as a key regulator of liver fibrosis and metabolism, the dynamic changes in BMP9 suggest that pulpitis may potentially affect patients’ systemic inflammatory status and metabolic health, offering insights for assessing the systemic risks of pulpitis and developing targeted intervention strategies. However, this study is primarily based on animal and cellular models and has not yet been validated in clinical populations. Additionally, the use of LPS from a single bacterial source fails to fully replicate the complex microbial environment in clinical settings, limiting the direct extrapolation of the findings. Future studies should incorporate clinical cohorts and multipathogen simulations to further validate its pathological and therapeutic implications. The concordance between the observed trends in pulpitis and systemic BMP9 levels suggested a plausible hypothesis: hepatic-derived BMP9, modulated by the systemic effects of pulpitis, might reach the site of pulp inflammation via the circulatory system, potentially exerting local regulatory effects. This hypothesis warrants further investigation to elucidate the precise mechanisms underlying this potential liver-pulp axis in the context of inflammatory processes.

## Conclusions

The present investigation demonstrated that LPS, a bacterial metabolite associated with pulpitis, could enter the systemic circulation and reach the liver. Upon arrival in hepatic tissue, LPS was observed to transiently upregulate levels of G9a and H3K9me2 in mHSCs, ultimately resulting in downregulated expression of BMP9. The findings of this study might provide valuable insights into the complex interplay between localized oral infections and systemic responses, potentially unveiling novel therapeutic targets for managing pulpitis and related inflammatory conditions.

## Author contributions

Tianzhu Song: Methodology, data curation, funding acquisition, and writing original draft. Dongzhe Song: Project administration, methodology, and writing review and editing. Yanglin Zeng: Methodology and data curation. Jingli Zhu: Validation and formal analysis. Xiangfen Li: Data curation, writing review and editing, and funding acquisition. Dingming Huang: Project administration, conceptualization, and supervision.

## Conflict of interest

The authors deny any conflicts of interest related to this study.

## Appendix Supplementary materials

Image, application 1
