# Supplementary material for: Pulpitis Transiently Affect Hepatic Bone Morphogenetic Protein 9 Expression by Lipopolysaccharide
Source: Int Dent J. 2026 Feb 19;76(2):109435. doi: 10.1016/j.identj.2026.109435 (PMC12933815; doi:10.1016/j.identj.2026.109435)
Supplement: Supplementary file 1 [file mmc1.doc]

**Supplementary Results:**


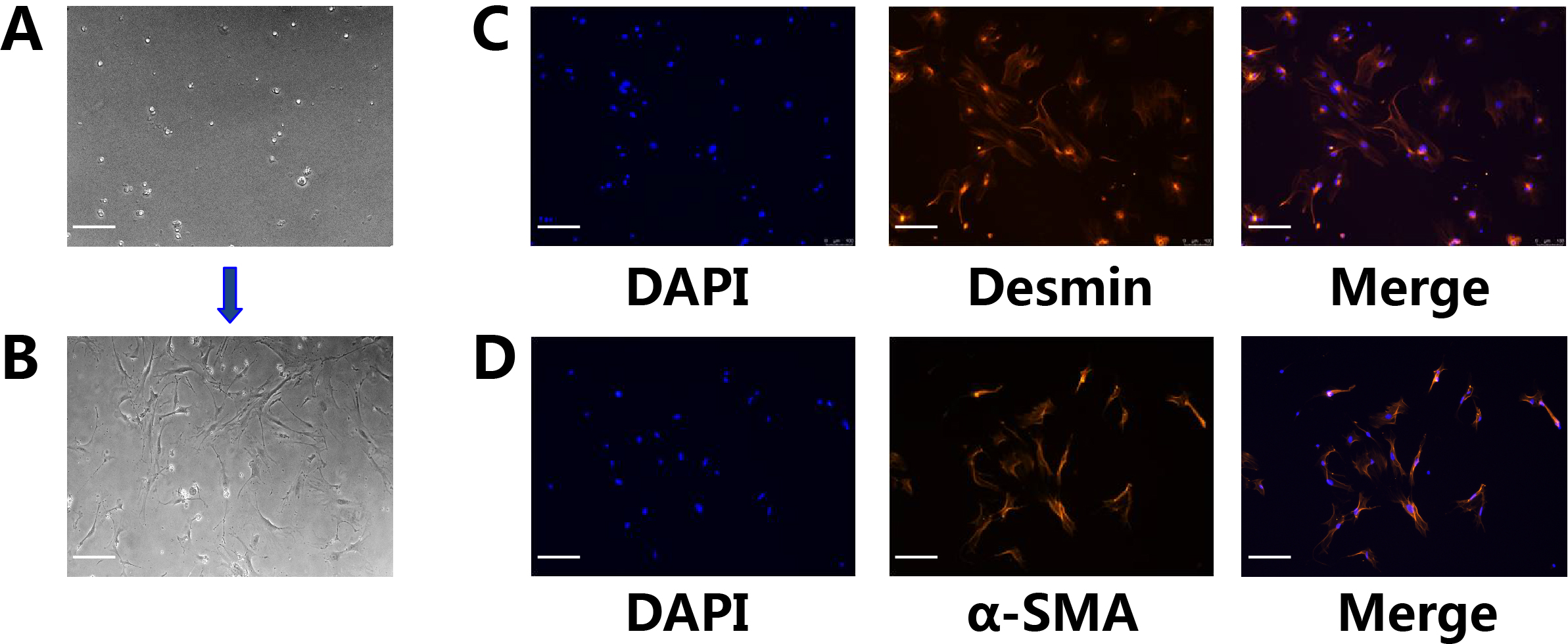


**Supplementary Figure 1.** The identification of mHSCs. Morphological identification: from the initial circle (A) to star, polygon (B) transformation. Cell markers detection : α-SMA (C), Desmin (D). Scale: 70 μm.


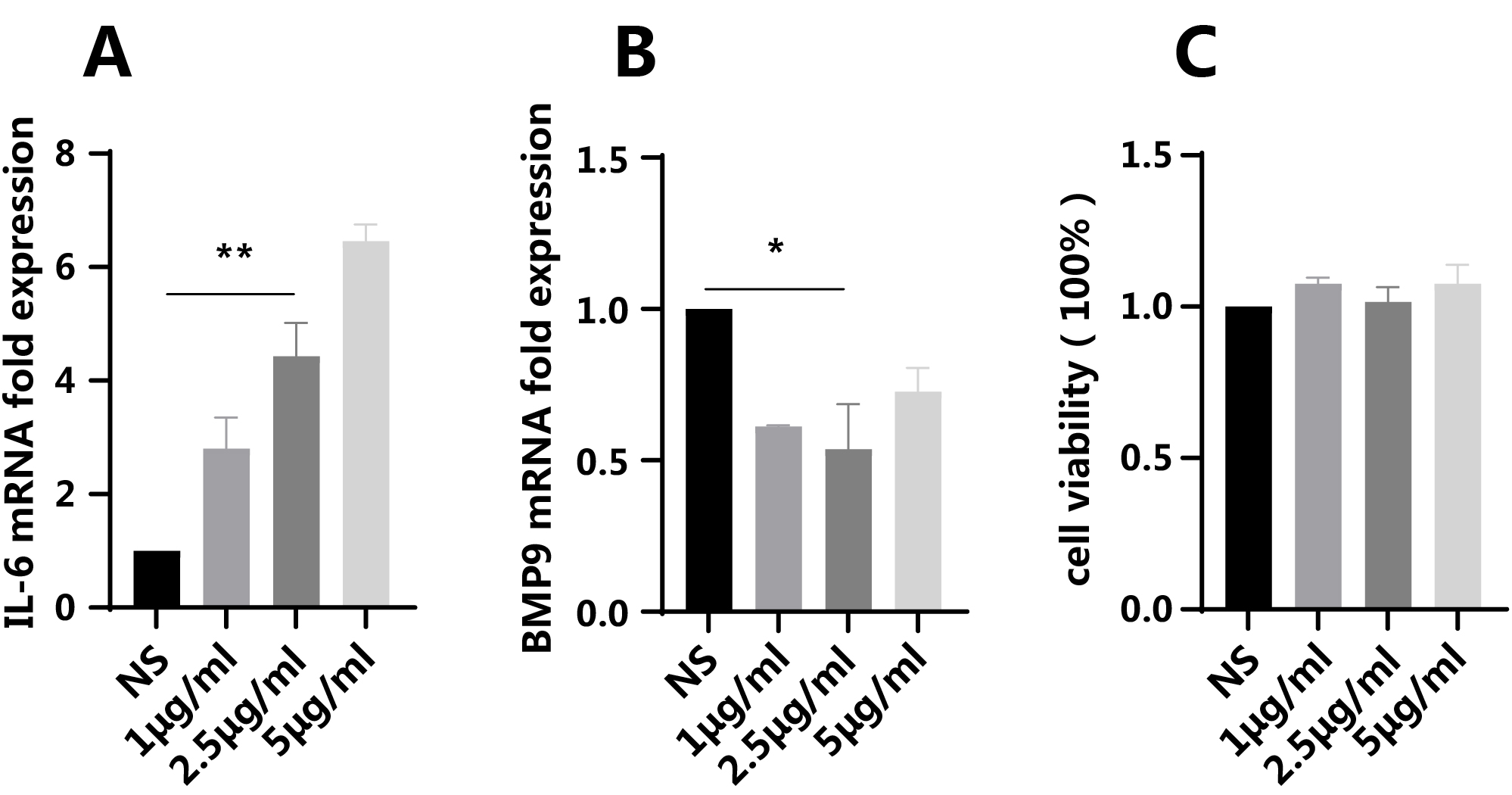


**Supplementary Figure 2.** *P.gingivalis* LPS concentration screening for stimulating mHSCs. **(A)** RT-qPCR results showed that IL-6 mRNA expression was upregulated with increased *P.gingivalis* LPS stimulation concentration (3h). **(B)** RT-qPCR results showed that the expression of BMP9 decreased most significantly at 2.5μg/mL *P.gingivalis* LPS concentration (3h). **(C)** The mHSCs activity did not change significantly at 48 h after *P.gingivalis* LPS stimulation at different concentrations (CCK8). Data are presented as mean ± SD (*n*=3). *P < 0.05. **P < 0.01.


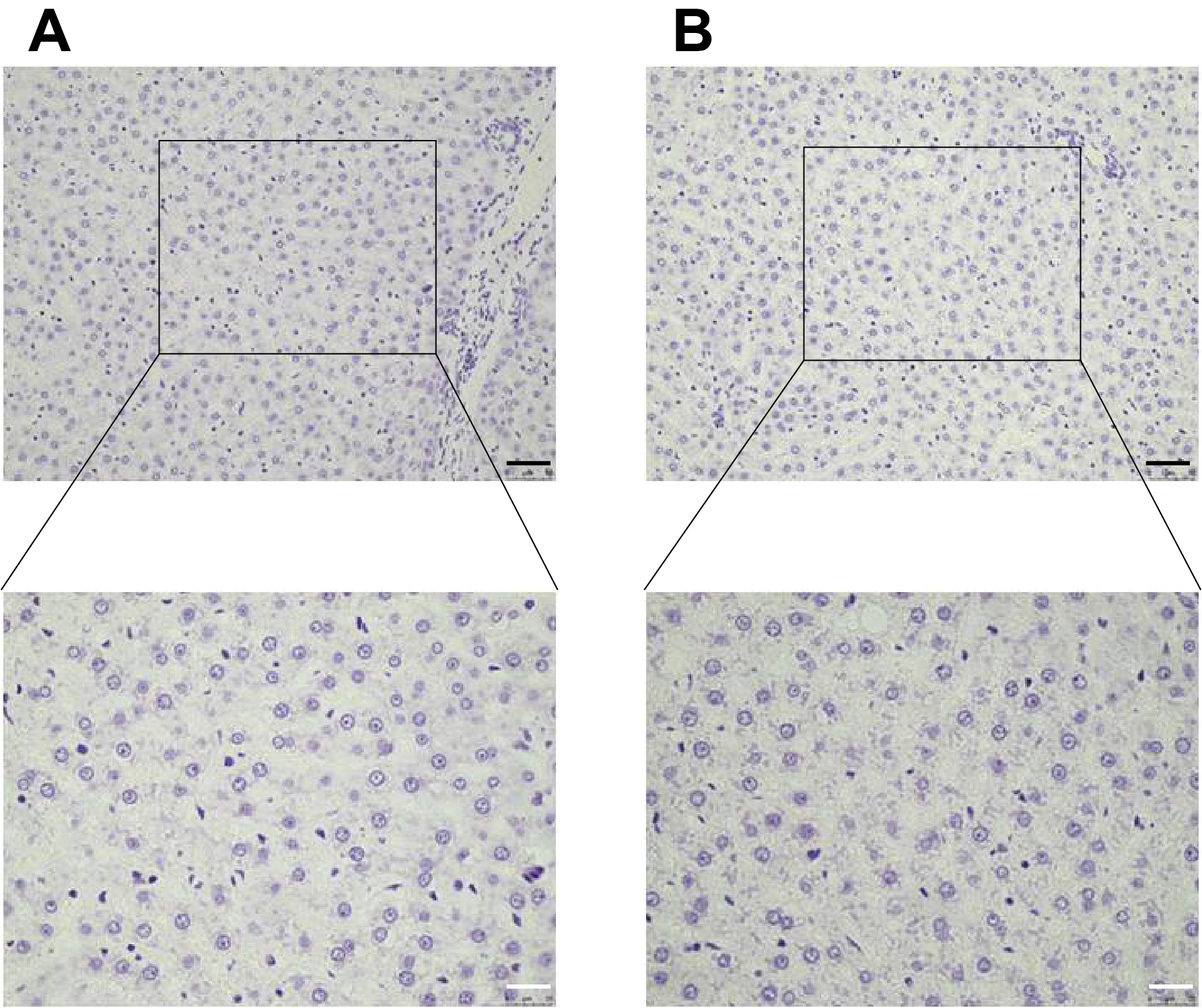


**Supplementary Figure 3.** The IHC control group without primary antibody in liver tissue. **(A)** Mouse liver tissue. **(B)** Rat liver tissue. Black scale: 50μm, white scale: 25μm.

**Supplementary Table 1**

**Primers**

| Gene | Forward (5’-3’) | Reverse (5’-3’) |
| --- | --- | --- |
| BMP9 | CGTCCAACATTGTGCGGAG | GACAGGAGACATAGAGTCGGAG |
| IL-6 | TGCAATAACCACCCCTGACC | GTGCCCATGCTACATTTGCC |
| GAPDH | CCAGAACATCATCCCTGCCT | CCTGCTTCACCACCTTCTTG |

**Supplementary Table 2**

**Primers for BMP9 promo**ter

| Gene | Forward (5’-3’) | Reverse (5’-3’) |
| --- | --- | --- |
| BMP9 1 | CTCTTTGAATGTGGAGGGTGTT | CTCTTTGAATGTGGAGGGTGTT |
| BMP9 2 | CATCTTTCCCAGTCCTGCTTC | TCCTATGAAGGCTGCTTGAGTT |
| BMP9 3 | AACCAAGTTGTCCTCTGAATACCC | GCTATGTTCAACCACCCTCCAG |
| BMP9 4 | TCAGCATCAGCACTGGAGGG | GAAAATGAGGCTGGGGAGTT |
| BMP9 5 | CGGGTTCCGATACCATAAGG | TCACGGTCTCCCCAACAATC |
